# Arsenic bioaccumulation in subarctic fishes of a mine-impacted bay on Great Slave Lake, Northwest Territories, Canada

**DOI:** 10.1371/journal.pone.0221361

**Published:** 2019-08-23

**Authors:** John Chételat, Peter A. Cott, Maikel Rosabal, Adam Houben, Christine McClelland, Elise Belle Rose, Marc Amyot

**Affiliations:** 1 Environment and Climate Change Canada, National Wildlife Research Centre, Ottawa, ON, Canada; 2 Environment and Natural Resources—Cumulative Impact Monitoring Program, Government of the Northwest Territories, Yellowknife, Canada; 3 Département des Sciences Biologiques, Université de Quebec à Montréal, Montreal, Canada; 4 Consultant, Ottawa, Canada; 5 Geography and Environmental Studies, Carleton University, Ottawa, Canada; 6 Centre d’Études Nordiques (CEN), Département de Sciences Biologiques, Université de Montréal, Montréal, Canada; CINVESTAV-IPN, MEXICO

## Abstract

A subarctic fish community in mine-impacted Yellowknife Bay (Great Slave Lake, Northwest Territories, Canada) was investigated for biological and ecological processes controlling arsenic bioaccumulation. Total concentrations of arsenic, antimony, and metals were measured in over 400 fishes representing 13 species, and primary producers and consumers were included to characterize food web transfer. Yellowknife Bay had slightly more arsenic in surface waters (~3 μg/L) relative to the main body of Great Slave Lake (<1 μg/L), resulting in two-fold higher total arsenic concentrations in muscle of burbot (*Lota lota*), lake whitefish (*Coregonus clupeaformis*), and northern pike (*Esox lucius*). Other mining-associated contaminants, specifically antimony, lead, and silver, were typically below analytical detection in those fish species. No evidence was found for enhanced bioaccumulation of arsenic in long-lived, slow-growing subarctic fishes. Food web biodilution of total arsenic occurred between primary producers, aquatic invertebrates, and fish, although trophic position did not explain arsenic concentrations among fishes. Pelagic-feeding species had higher total arsenic concentrations compared to littoral fishes. Arsenic accumulated in subarctic fishes to comparable levels as fishes from lakes around the world with similar water arsenic concentrations. This first comprehensive study for a subarctic freshwater food web identified the importance of water exposure, biodilution, and habitat-specific feeding on arsenic bioaccumulation.

## Introduction

Arsenic is a toxic element that negatively impacts fish health at high levels of exposure [1]. Elevated arsenic has been shown to reduce fish growth, inhibit feeding, cause lesions in tissues such as liver, and reduce egg production [2–5]. This non-essential element accumulates in fish following uptake from water and diet [6–8]. Dietary uptake of arsenic is likely more important than absorption directly through water unless water concentrations are extremely high [7, 9]. The assimilation efficiency of dietary arsenic and its retention in fish tissues is low, resulting in rapid efflux rates [6, 9, 10]. Arsenic speciation in the diet affects the degree of toxicity to fish, with inorganic arsenic having higher toxicity than some organoarsenicals [11]. In addition, fish species vary in their capacity to metabolize inorganic arsenic and transform it into more benign organic species [12].

Fish species inhabiting a contaminated waterbody can differ considerably in their tissue arsenic concentration, and the underlying environmental processes driving species-specific bioaccumulation are not clear [13–15]. Biological factors (e.g., species, size, age) and ecological factors (e.g., diet sources, trophic position) may influence the accumulation of arsenic in fishes [1, 16]. The role of body size is not consistent in the literature, with observations of positive, negative, and no correlation between body size and arsenic concentrations documented in studies of freshwater fishes [14, 17–19]. Arsenic speciation in the diet (i.e. inorganic versus organic forms) and differences among fish species in their physiological capacity to process arsenic are additional determinants of bioaccumulation in fishes [12, 20]. Biodilution occurs during trophic transfer of arsenic in aquatic food webs (rather than biomagnification) [21–23], but cases of greater arsenic concentrations in fishes of higher trophic position have been reported [14, 24]. Feeding habitat may be important; planktivorous fishes in temperate lakes can accumulate more arsenic than those feeding in other habitats [18, 21]. To our knowledge, arsenic accumulation in Arctic freshwater food webs has not been characterized, and more information is needed on food web responses to point-source exposure of arsenic in northern lakes.

**Fig 1:**
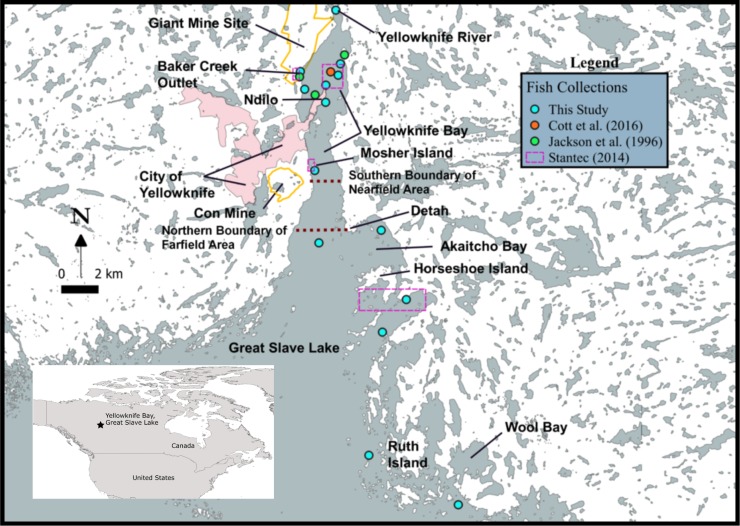
Map of the study area on Great Slave Lake. Inset map shows the location of Great Slave Lake and the Northwest Territories within Canada. Boundaries between the nearfield and farfield areas in Yellowknife Bay are identified as well as approximate fish collection sites for this study and literature data. Locations are presented for the Giant and Con mine sites and communities (Yellowknife, Ndilo and Detah) within the study area.

Metal mining and smelting are significant point sources of arsenic that can highly contaminate local environments [5, 13, 22, 25, 26]. In subarctic Canada, gold production at Yellowknife (Northwest Territories) resulted in extensive environmental contamination of arsenic. Two main operations, the Giant and Con mines, were in production from the late 1930s or 1940s to the early 2000s [27], during which an estimated 20,000 tonnes of arsenic trioxide were emitted to the air from the ore roaster stack at Giant Mine [28]. Lesser amounts of arsenic were also released from the Con Mine roaster stack until 1970 [29]. Yellowknife Bay (Fig 1) received loadings of arsenic, antimony, and metals via tailings dumps and mine effluent from the Giant Mine, as well as atmospheric deposition from ore roasting emissions at Giant and Con mines [30, 31].

Early environmental study in the 1970s indicated that Giant Mine effluent entering Yellowknife Bay was acutely toxic to fish [32] and likely had a negative impact on the benthic invertebrate community [32, 33]. An effluent treatment plant was subsequently constructed to reduce arsenic, antimony, and metal loadings to the bay. More recent measurements of total arsenic were conducted on large-bodied fish species, as well as the speciation of arsenic in their tissues [34–37]. Site and species variation of total arsenic concentrations were identified through those environmental studies, but there has been no examination of processes affecting arsenic bioaccumulation and food web transfer.

Yellowknife Bay and adjacent areas in Great Slave Lake are important for recreational and subsistence harvesting of fishes, as well as a local commercial fishery. Contamination of the fish community of Yellowknife Bay remains an issue of concern despite the closure of Giant and Con mines in the early 2000s [38], and the main objective of this study was to characterize the processes controlling fish exposure to arsenic in the mine-impacted area. Data were generated from field collections in 2013 to 2015 and combined with grey literature data from other environmental studies of Yellowknife Bay to conduct a comprehensive analysis. Total arsenic concentrations in fishes were compared with those of other metal(loid)s released from mining activities to examine the relative bioaccumulation of those elements in the ecosystem. Spatial and temporal variation in fish exposure to arsenic were determined for the study area. The roles of biological factors (species, size, age, tissue-specific accumulation) were examined as well as food web influences (trophic position, habitat-specific feeding) on total arsenic accumulation using carbon and nitrogen stable isotopes. Finally, total arsenic concentrations in fishes of other arsenic-contaminated water bodies around the world were compared to the bioaccumulation response in this subarctic study area. The findings, generated from a large dataset of over 400 fishes representing 13 species, advance our understanding of environmental processes controlling the food web accumulation of arsenic in mine-impacted lakes.

## Materials and methods

### Study area

Yellowknife Bay is located in the North Arm of Great Slave Lake (Fig 1), the deepest lake in North America (maximum depth = 614 m) and the ninth largest lake in the world by surface area (28,568 km^2^) [39]. This subarctic region of the Northwest Territories has a mean annual precipitation of 289 mm and a daily average temperature of 17°C in July and -26°C in January [40]. Great Slave Lake is ice covered for approximately seven months of the year from November to June. The lake supports a diverse fish community of over 25 species typical to large, cold-water oligotrophic lakes in northern Canada [41]. Yellowknife Bay has a surface area of approximately 20 km^2^ with a maximum water depth of 15 m at the north end and 30 m at the south end, where it meets the main body of Great Slave Lake [42]. The Yellowknife River, which enters the north end of Yellowknife Bay, is the main fluvial source of water and is an important spawning and migration corridor for species of *Coregonus* [43].

Arsenic and antimony, and to a lesser extent copper, lead, manganese, mercury, silver, and zinc were the most highly enriched metal(loid)s released by mining activities at the north end of Yellowknife Bay [30]. Surface water arsenic (post-mine closure) is relatively low near the Giant Mine at the north end of the bay (generally < 5 μg/L), but still above background concentrations (< 1 μg/L) [30]. Maximum contamination in sediment declines exponentially with distance south of Giant Mine in Yellowknife Bay and is greatest within 5 km of the mine [30]. However, lower contamination in sediment occurred as far as 25–30 km south of the mine into the main body of Great Slave Lake. Based on this delineation of contamination by Chételat et al. [30], fish were grouped into one of two study areas according to location of capture: the nearfield (Yellowknife Bay north of Mosher Island, including Back Bay and the mouth of Baker Creek) or the farfield (the mouth of Yellowknife Bay near Detah, and the main body of Great Slave Lake, including Akaitcho Bay, Horseshoe Island, Ruth Island, and the Wool Bay area) (Fig 1). Two juvenile fish captured at the mouth of the Yellowknife River were included in the farfield fish group because the site was upstream of Giant Mine. Greater exposure to mining contamination was anticipated for fishes collected in the nearfield area compared to the farfield.

### Fish collection

A total of 176 fish or composite samples of 13 species were collected over three years between August and October of 2013, 2014 and 2015. Multiple capture methods (multi-panel gill nets with variable mesh size of 1–3.5 inches, baited long lines, shoreline electrofishing) were used to collect a variety of fish species and a wide size range for three dominant species. The most commonly collected species were burbot (*Lota lota*, *n* = 53), lake whitefish (*Coregonus clupeaformis*, *n* = 43), and northern pike (*Esox lucius*, *n* = 38). Other species collected were longnose sucker (*Catostomus catostomus*, *n* = 13), cisco (*Coregonus artedi*, *n* = 10), slimy sculpin (*Cottus cognatus*, *n* = 2 individuals, *n* = 4 pools of 2–5 fish), round whitefish (*Prosopum cylindraceum*, *n* = 4), lake trout (*Salvelinus namaycush*, *n* = 2), shiners (*Notropis* spp., *n* = 2 pools of ~50 fish), walleye (*Sander vitreus*, *n* = 2), inconnu (*Stenodus leucichthys*, *n* = 1), ninespine stickleback (*Pungitius pungitius*, *n* = 1 pool of 8 fish), and white sucker (*Catostomus commersonii*, *n* = 1). The general locations of fish capture sites are identified in Fig 1.

Individual fish were measured for length and weight, bone structures (otoliths or cleithra of northern pike) were removed for ageing, and the liver and a dorsal muscle sample were removed for chemical analysis. Small-bodied fish species, specifically slimy sculpin, ninespine stickleback, and shiners were analyzed as whole-body composite samples of multiple individuals. Juvenile burbot and northern pike (< 80 g body mass, *n* = 15) were analyzed as whole body samples of individual fish. Ageing structures were removed from juvenile burbot, lake whitefish, and northern pike but not from other small-bodied fish. Fish samples were frozen at -20°C until lab processing. Fish ages were estimated by Aqua-Tech Services (Perth, Ontario) using standard protocol for cleithra of northern pike or the crack and burn method for otoliths.

### Aquatic food web

Benthic primary producers and aquatic invertebrates were sampled to measure element concentrations at the base of the food web. Plankton were collected by vertical hauls through the entire water column using two nets of different mesh size (64 μm and 200 μm). These bulk samples contained a mix of phytoplankton and zooplankton. Thick biofilm mats and aquatic plants (macrophytes) were sampled along shoreline areas by hand with gloves. Offshore sediment was collected with an Ekman grab and sieved (500 μm mesh size) to obtain profundal amphipods. Amphipods were depurated in ultrapure water for 48 hours. Aquatic biota were frozen at -20°C until laboratory processing.

### Laboratory analyses

Fish tissue, amphipods, plankton, and benthic primary producers were freeze-dried for 48 hours and homogenized prior to chemical analysis. Small-bodied fishes were homogenized whole-body with a grinder prior to freeze-drying. In order to develop whole-body to muscle conversion factors for element concentrations, a random sub-sample of five cisco and five juvenile lake whitefish were dissected for chemical analysis of both the whole-body and muscle. For each of those ten fish, a small muscle sample was removed and weighed. The remaining carcass was then reweighed and processed as a whole-body sample. Whole-body element concentrations for those ten fish were corrected for partial muscle removal using a mass balance calculation as per Chételat et al. [44].

A total of 250 fish samples (152 muscle, 64 liver, 34 whole body individuals or composites) and 41 lower trophic food web samples (16 plankton, 15 amphipod, 7 rock biofilm, 3 macrophyte) were analyzed for a suite of elements by microwave-assisted digestion in nitric acid and detection on an inductively coupled plasma mass spectrometer (ICP-MS). This study focused on the results of seven priority elements: antimony, arsenic, copper, lead, manganese, silver, and zinc. The majority of analyses were conducted at RPC Laboratories (Fredericton, New Brunswick) for fish and most food web samples, although some of the fish collected in the first year (2013) were analyzed at Innotech Alberta (formerly Alberta Innovates Technology Futures, Vegreville, Alberta) and profundal amphipods were analyzed for arsenic at the Laboratory of Environmental Biogeochemistry (Université de Montréal, Montreal, Quebec). Analytical blanks were consistently below detection for the elements of interest. The relative standard deviation (RSD) of 24 duplicate digestions of fish samples averaged <10% for the elements of interest (mean RSD ± SD for arsenic = 4 ± 9%, copper = 5 ± 11%, manganese = 6 ± 10%, silver = 5 ± 7%, zinc = 3 ± 8%). RSDs were not calculated for antimony and lead because most duplicate results were below analytical detection. Mean (± SD) recoveries of analytes from fish certified reference materials of the National Research Council of Canada (DORM-4: *n* = 15, DOLT-4: *n* = 6, and DOLT-5: *n* = 8) were as follows: arsenic = 91 ± 6%, copper = 98 ± 7%, lead = 104 ± 11%, manganese = 97 ± 4% (DORM-4 only), silver = 88 ± 9%, and zinc = 88 ± 6%.

Fish (muscle or whole-body) and food web samples collected in this study were analyzed for carbon and nitrogen stable isotopes at the GG Hatch Stable Isotope Laboratory (University of Ottawa, Ottawa, Ontario). Freeze-dried and homogenized material (1 mg) was analyzed on a Delta Advantage Isotope Ratio Mass Spectrometer interfaced by a Conflo III to a Vario EL Cube elemental analyzer. Stable isotope ratios were expressed in delta notation (δ) as the parts per thousand (‰) deviation from standards of atmospheric N_2_ gas for δ^15^N and Vienna PeeDee Belemnite for δ^13^C.

### Data analysis

#### Dataset of element concentrations in fishes

Data from the 2013–2015 fish collection, including size and age measurements, ICP-MS total element concentrations, and carbon and nitrogen stable isotope ratios were made publicly available on the Dryad Data Repository (https://doi.org/10.5061/dryad.c2q52rs). Whole-body element concentrations of small-bodied fishes (*n* = 24) were converted to muscle concentrations using measured factors for the ratio of muscle:whole-body concentration (details in S1 Text).

Fish data generated in this study were combined with total element concentrations in fishes of the study area reported in the grey literature and a published paper to conduct a more comprehensive analysis of metal(loid) accumulation in fish. Fish size, age, and element concentrations in muscle and liver were included from: Stantec [35] for 7 burbot, 84 lake whitefish, and 104 northern pike captured in 2012–2013, Cott et al. [36] for 7 burbot and 17 lake whitefish captured in 2011, and Jackson et al. [34] for 11 burbot, 128 lake whitefish, and 21 northern pike captured in 1992–1993. Additional detail on the compilation of these datasets is provided in S1 Text.

#### Comparison of element concentrations in fishes

Means and ranges (minimum-maximum values) of total concentrations for priority elements (antimony, arsenic, copper, lead, manganese, silver, zinc) are reported for 142 northern pike, 144 lake whitefish and 67 burbot using the data from this study, Cott et al. [36], and Stantec [35]. Multivariate canonical redundancy analysis (RDA) was conducted to examine the importance of location (nearfield vs. farfield), species, tissue type (liver vs. muscle), fish size (length and mass), and fish age for metal(loid) bioaccumulation. The response variables (concentrations of elements above analytical detection) were log-transformed and centered, while continuous explanatory variables were centered and scaled. The RDA was performed with R statistical package [45] using the vegan library [46]. The ordistep function was used to identify statistically significant environmental or biological explanatory variables.

#### Influence of spatial and biological factors

The influence of size (length, mass) and age on log-transformed total arsenic concentrations in liver and muscle was examined by Pearson correlation analysis. Probability values were adjusted for the experiment-wise error rate using Holm’s correction. General linear models (GLMs) were used to test the effect of capture location (nearfield vs. farfield) on log-transformed arsenic concentrations in fish and were performed with the linear model function in R. Fish length was included as a covariate (when a significant variable) to account for different fish sizes between areas. GLMs were conducted separately for each species and tissue type because of the variable correlations with fish size. GLM results were determined using type III sums of squares with the car package [47].

#### Temporal comparison of fish arsenic concentrations

Data from Jackson et al. [34] were used to investigate temporal change in log-transformed liver and muscle total arsenic concentrations of burbot, lake whitefish, and northern pike captured in 1992–1993 with those captured approximately 20 years later in 2011–2015. The Giant Mine was producing gold on site until 1999, and the two time periods reflect conditions before and after mine closure in 2004. Only fishes from the nearfield area were examined. GLMs were used to test for differences in tissue total arsenic concentrations between the two time periods, with the inclusion of fish length as a covariate in the model (when *p* < 0.05).

#### Food web accumulation and trophic transfer of arsenic

Data from benthic primary producers, amphipods, plankton, and fish from 13 species were pooled to examine the influence of food web structure on total arsenic bioaccumulation using carbon and nitrogen stable isotopes. Stable isotope measurements were not available for fishes from Stantec [35] and Cott et al. [36]. Trophic transfer of arsenic was tested by GLMs of log-transformed total arsenic concentration (muscle or liver) as the dependent variable and trophic position (estimated with δ^15^N values), capture area, and a capture area*δ^15^N interaction as the independent variables. The influence of habitat-specific feeding was similarly modelled using δ^13^C values, which vary for fishes feeding on carbon from littoral or pelagic habitats [48].

#### Comparison of arsenic bioaccumulation in Great Slave Lake with fishes from other studies

A literature search was performed using the abstract and citation database Scopus (Elsevier B.V.) to compile and compare published field studies around the world where total arsenic concentrations were measured in both water and fishes of lakes or ponds. Water arsenic concentration in each waterbody was used as an indicator of ecosystem exposure to the food chain. The dataset consisted of 67 records of average total arsenic concentrations in fish muscle (μg/g dry weight) from 33 waterbodies, which were obtained from ten published papers [13, 15, 18, 23, 49–54], a consultant report for an adjacent lake (Lower Martin Lake) impacted by the Giant Mine [35], and this study. Where possible, mean concentrations of individual fish species from the same waterbody were included as separate data entries. Additional detail on the global water-fish arsenic dataset is provided in S1 Text.

## Results

### Element concentrations in burbot, lake whitefish and northern pike

**Fig 2:**
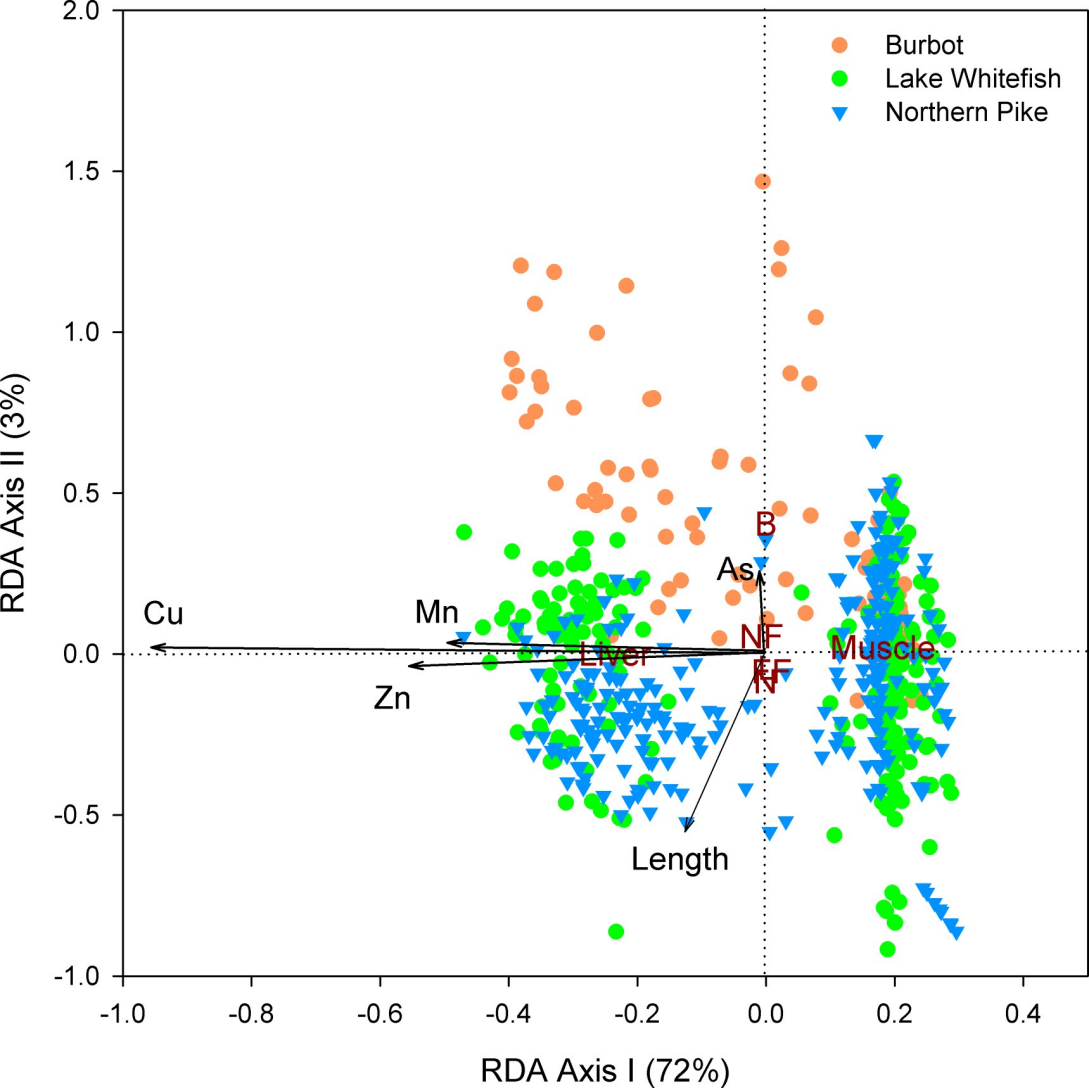
RDA correlation triplot of metal(loid)s in fishes of Great Slave Lake, Northwest Territories, Canada. Total concentrations of arsenic (As), copper (Cu), manganese (Mn) and zinc (Zn) are presented as a function of species (B = burbot, L = lake whitefish, N = northern pike), tissue type, capture area (NF = nearfield, FF = farfield), and fish length. The RDA model was highly significant (*F* = 360, *p* < 0.001, *n* = 572), and the first 2 canonical axes explained 75% of the variation of element concentrations in fishes.

Total concentrations of arsenic, antimony, and metals in liver and muscle of northern pike, lake whitefish and burbot are presented in Table 1. Antimony and lead were consistently below analytical detection in both tissue types and all three species. Silver was below analytical detection in muscle but not liver. Multivariate RDA indicated that concentrations of detectable study metal(loid)s (arsenic, copper, manganese, zinc) in 572 fish samples were explained primarily by tissue type (*F* = 1703, *p* < 0.005) and to a lesser extent by fish species (*F* = 35, *p* < 0.005), area (*F* = 30, *p* < 0.005), and fish length (*F* = 21, *p* < 0.005) (Fig 2). Fish age and mass were not significant explanatory variables (*p* ≥ 0.08) and were excluded from the RDA model. Copper, manganese, and zinc showed high covariance associated with the first RDA axis. Liver had higher concentrations of those three elements compared with muscle. In contrast, arsenic was associated with the second RDA axis, with a tendency to be higher in burbot than in northern pike or lake whitefish (Fig 2). Although area was a significant explanatory variable in the RDA model, the proximity of the centroids for this factor indicated little overall difference in metal(loid) concentrations in fishes between the nearfield and farfield (Fig 2).

**Table 1:** Metal(loid) total concentrations in liver and muscle of burbot, lake whitefish, and northern pike collected in Great Slave Lake, Northwest Territories, Canada.

			Burbot		Lake Whitefish			Northern Pike		
		*n* = 67 (M), 49 (L)			*n* = 144 (M), 100 (L)			*n* = 142 (M), 124 (L)		
Element	Tissue	Mean	Range	% b.d.	Mean	Range	% b.d.	Mean	Range	% b.d.
Antimony	Muscle	b.d.	<0.02–13.4	84	b.d.	<0.02	100	b.d.	<0.02–0.35	94
	Liver	b.d.	<0.02–0.03	88	b.d.	<0.05	100	b.d.	<0.02–0.65	94
Arsenic	Muscle	2.9	0.5–21.6	0	0.7	<0.2–2.1	5	0.9	0.2–3.2	0
	Liver	3.0	0.8–9.2	0	0.9	<0.25–2.9	1	0.5	<0.25–2.0	6
Copper	Muscle	2.1	0.6–10.6	0	1.1^¥^	<2.5–5.6	57	1.3^¥^	<2.5–4.4	65
	Liver	46	4.4–146	0	46	8.1–258	0	29	<2.5–140	6
Lead	Muscle	b.d.	<0.02–0.45	78	b.d.	<0.02–0.25	92	b.d.	<0.02–0.06	96
	Liver	b.d.	<0.02–0.38	96	b.d.	<0.02–2.28	95	b.d.	<0.02–0.04	96
Manganese	Muscle	2.7	0.5–23.2	0	0.8^¥^	<1.5–3.8	57	0.9^¥^	<1.5–3.9	68
	Liver	2.6	0.4–8.4	0	7.5	3.9–15.0	0	3.9	<1.5–9.8	6
Silver^β^	Muscle	b.d.	<0.02–0.02	98	b.d.	<0.02	100	b.d.	<0.02–0.03	99
	Liver	0.2	<0.02–0.6	2	0.4^¥^	<0.25–2.9	40	0.3^¥^	<0.02–1.9	38
Zinc	Muscle	25.7	13.4–91	0	16.2	9.0–69	0	21.5	13.2–70	0
	Liver	71	18.7–162	0	146	87–491	0	151	<10–933	6

Concentrations are presented on a dry weight basis as μg/g. Fishes were collected in the study area from 2011 to 2015. Mean concentrations were not presented for tissues that had > 75% of results below analytical detection for a given element. Half the detection limit value was used when a lower proportion of results were below detection. In cases of more than 1 detection limit for an element, the lowest detection limit is presented for the range.

M = muscle, L = liver, b.d. = below detection

^β^Sample sizes are lower for silver, which was not analyzed for fishes from Cott et al. [36]

^¥^Average value is an estimate due to large number of results below analytical detection

**Fig 3:**
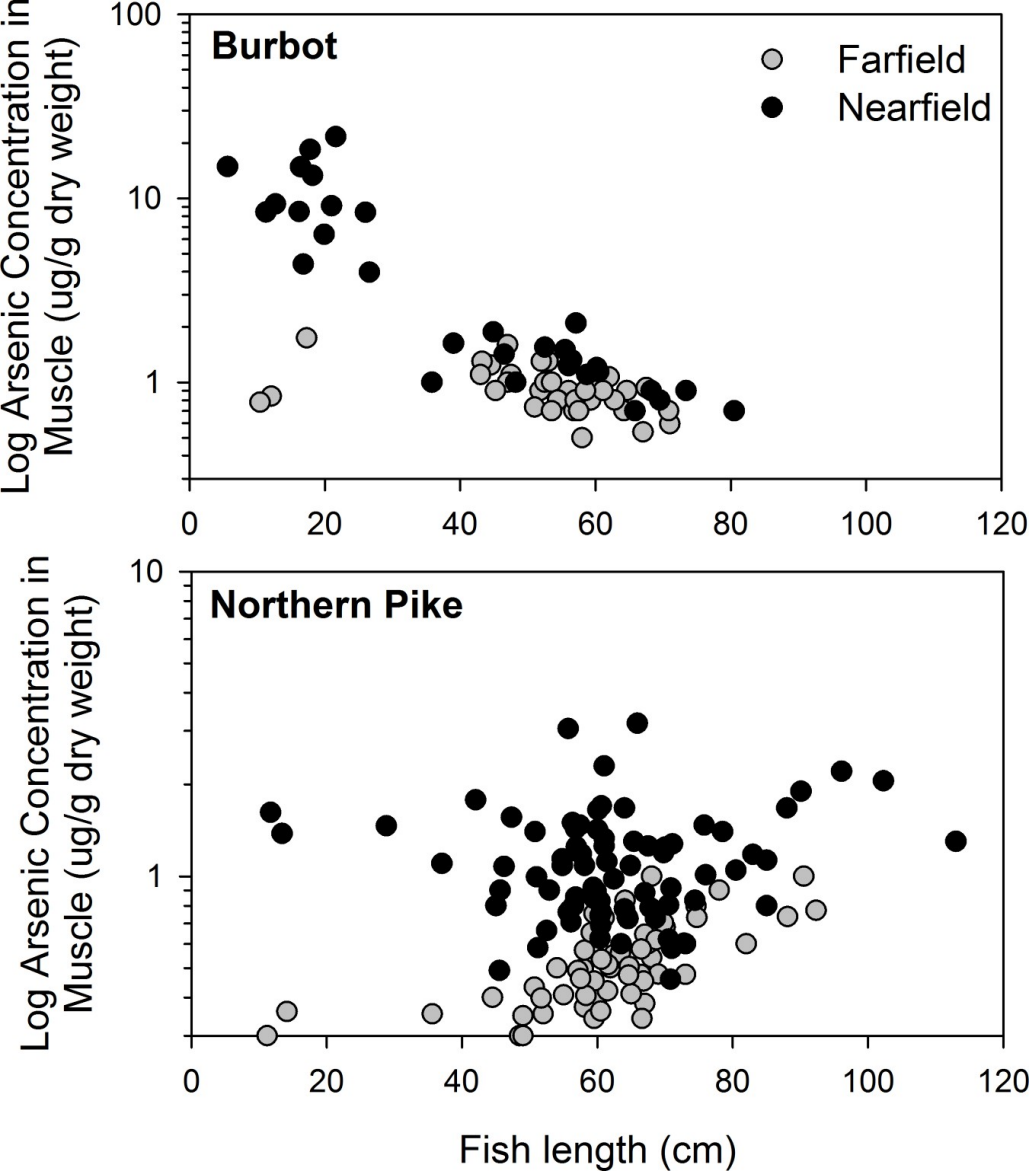
Correlations of total arsenic and length of burbot or northern pike differ between the nearfield and farfield. Muscle total arsenic concentrations are presented on a logarithmic scale, and correlations (pooled nearfield and farfield data) are statistically significant (burbot: Pearson *r* = -0.78, *p* < 0.001, *n* = 67; northern pike: Pearson *r* = 0.28, *p* < 0.01, *n* = 142). Data are identified by location of capture (nearfield or farfield) in Great Slave Lake, Northwest Territories, Canada.

The influence of size and age on total arsenic concentrations in fish tissues was examined in more detail by Pearson correlation analysis. Means and ranges of length, mass and age for northern pike, lake whitefish and burbot are summarized in Table 2. For burbot, total arsenic concentrations were negatively correlated with size and age in muscle but not in liver (Table 3). For lake whitefish, liver and muscle total arsenic concentrations were negatively correlated with size but positively correlated with age. For northern pike, total arsenic concentrations were positively correlated with size (muscle only) and not related to age. Inconsistent patterns of total arsenic concentrations in relation to fish size and age may reflect a confounding influence of differing arsenic exposure among size classes between nearfield and farfield areas, and/or species-specific responses (Fig 3). For burbot, a strong negative correlation between muscle total arsenic concentration and length was observed in the nearfield but not the farfield area. For northern pike, a positive correlation was found for farfield fish but not those captured in the nearfield area.

**Table 2:** Size and age of burbot, lake whitefish and northern pike collected from Great Slave Lake, Northwest Territories, Canada.

	Burbot			Lake Whitefish			Northern Pike		
Variable	Median	Range	*n*	Median	Range	*n*	Median	Range	*n*
Length (cm)	53	6–81	67	44	12–53	144	61	11–113	142
Mass (g)	900	7–3,150	67	860	14–1,840	144	1360	9–11,339	142
Age (years)	11	1–22	53	11	2–26	126	7	<1–17	142

**Table 3:** Pearson correlations of muscle and liver total arsenic concentrations in relation to fish length, mass, and age.

Tissue	Species	Length	Mass	Age
Muscle	Burbot	-0.78***	-0.63***	-0.64***
Muscle	Lake Whitefish	-0.25*	-0.26**	0.32***
Muscle	Northern Pike	0.28**	0.28**	0.18
Liver	Burbot	-0.17	-0.28	0.17
Liver	Lake Whitefish	-0.47***	-0.43***	0.34*
Liver	Northern Pike	-0.10	-0.14	-0.09

Sample sizes for correlation coefficients ranged from 42–144. Probability values were Holms-corrected. Concentrations were log-transformed.

* *p* < 0.05

** *p* < 0.01

*** *p* < 0.001

**Fig 4:**
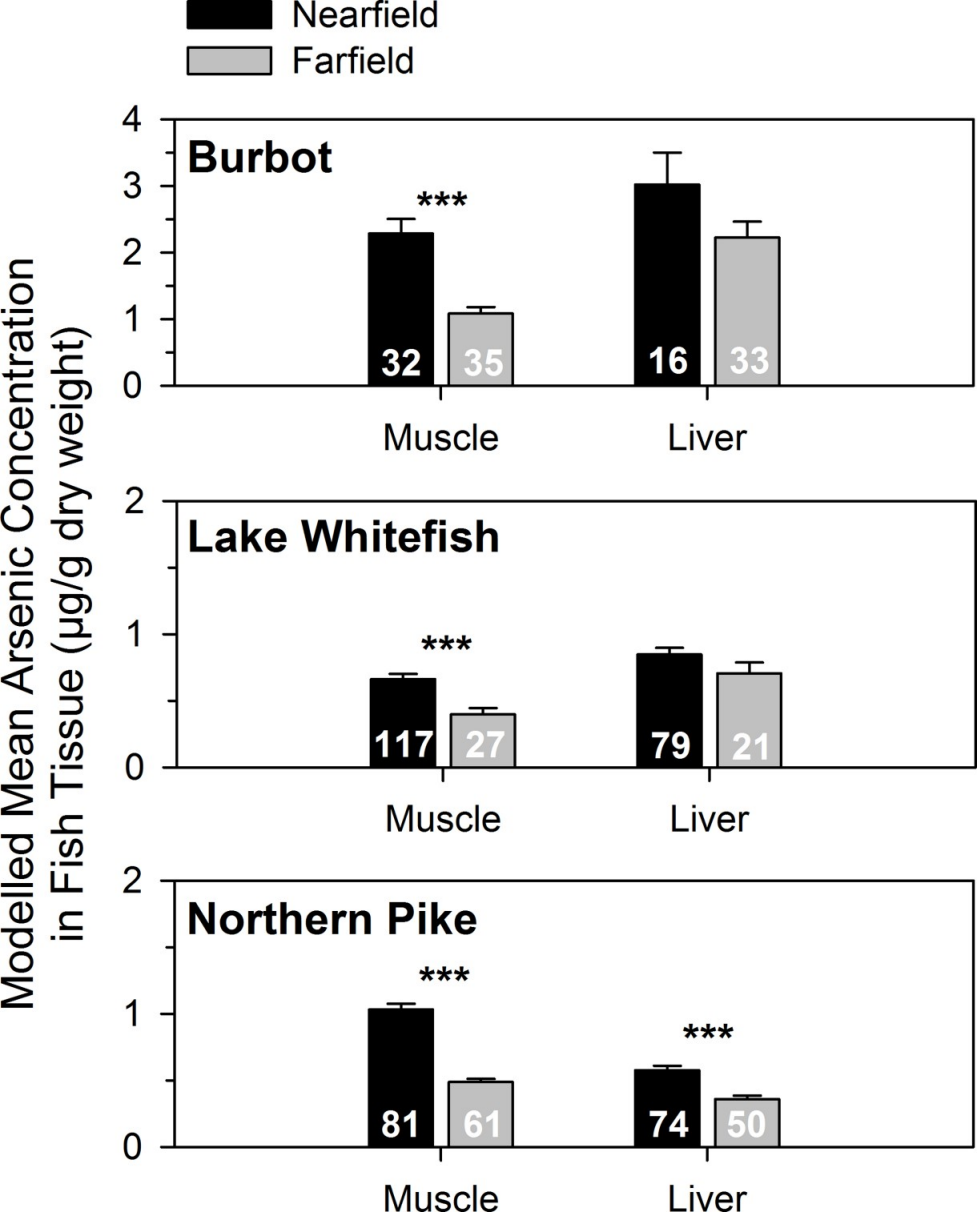
Comparison of total arsenic concentrations in fishes between the nearfield and farfield. Length-corrected mean estimates (anti-log, ± 1 standard error) of muscle and liver total arsenic from GLMs are presented (if length was a significant covariate) for burbot, lake whitefish, and northern pike of Great Slave Lake, Northwest Territories, Canada. Note the difference in y-axis scale between panels. Statistically significant differences are identified with asterisks (*** *p* < 0.001). Sample sizes are presented at the base of the bars.

Total arsenic concentrations were higher in fishes captured in the nearfield compared with the farfield area, although tissue-specific differences were observed (Fig 4). Length-adjusted total arsenic concentrations in muscle were significantly higher in the nearfield area for all three species (results for the factor “capture location” in the GLMs: *F* = 16–138, *p* < 0.001, *n* = 67–144). In contrast, liver total arsenic concentrations were significantly higher in northern pike captured in the nearfield area (*F* = 29, *p* < 0.001, *n* = 142) but not for burbot or lake whitefish (*p* ≥ 0.096, *n* = 49–100). Length-adjusted total arsenic concentrations were 2-fold higher in muscle and 1.4-fold higher in liver for fishes in the nearfield compared with the farfield (Fig 4).

There was species-specific variation in arsenic accumulation. Burbot had the highest concentrations of total arsenic in both muscle and liver, irrespective of the area of capture, whereas northern pike and lake whitefish had lower and similar levels of total arsenic in liver and muscle (Table 1, Fig 4). The distribution of total arsenic between muscle and liver also differed among fish species. Higher concentrations were observed in liver than muscle for burbot (mean ratio ± SE of liver:muscle arsenic = 2.9 ± 0.3) and lake whitefish (mean ratio = 1.8 ± 0.2), whereas northern pike had more total arsenic in muscle than liver (mean ratio = 0.7 ± 0.03). The highest concentrations of total arsenic in muscle were in some fish captured in north Yellowknife Bay (nearfield) at or near the Baker Creek outlet where arsenic-rich effluent flows into the bay. A total of 13 burbot were collected from that site, with higher mean total arsenic concentrations in muscle (mean ± SD = 10.9 ± 5.4 μg/g) compared to other burbot from the rest of the nearfield (1.2 ± 0.4 μg/g, *n* = 18).

### Temporal variation of fish arsenic concentrations

**Fig 5:**
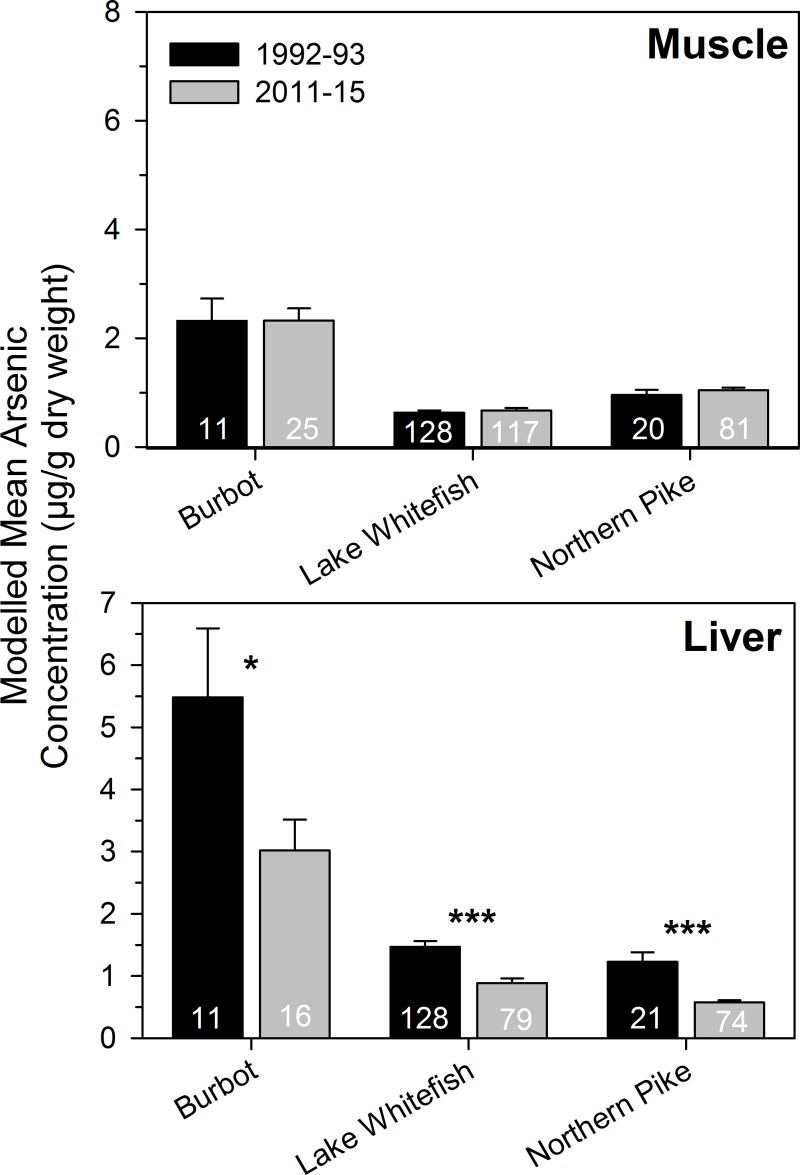
Comparison of total arsenic concentrations in fishes collected in 1992–93 and 2011–15. Length-corrected mean estimates (anti-log, ± 1 standard error) of muscle and liver total arsenic from GLMs are presented (if length was a significant covariate) for burbot, lake whitefish, and northern pike from the nearfield area of Yellowknife Bay, Great Slave Lake, Northwest Territories, Canada. Statistically significant differences are identified with asterisks (* *p* < 0.05, *** *p* < 0.001). Sample sizes are presented at the base of the bars.

There was some evidence of lower total arsenic accumulation in more recently collected fishes from the nearfield area compared to a collection two decades earlier in 1992–1993 (Fig 5). Liver total arsenic concentrations were significantly lower (by ~50%) in the more recent collection (2011–2015) for burbot, lake whitefish, and northern pike (effect of collection period in GLMs: *F* = 6–32, *p* = 0.019–<0.001, *n* = 27–207). However, there was no difference in muscle total arsenic concentrations between collection periods for any of the fish species (*F* < 1, *p* > 0.425, *n* = 42–245).

### Trophic transfer of arsenic in the food web

**Fig 6:**
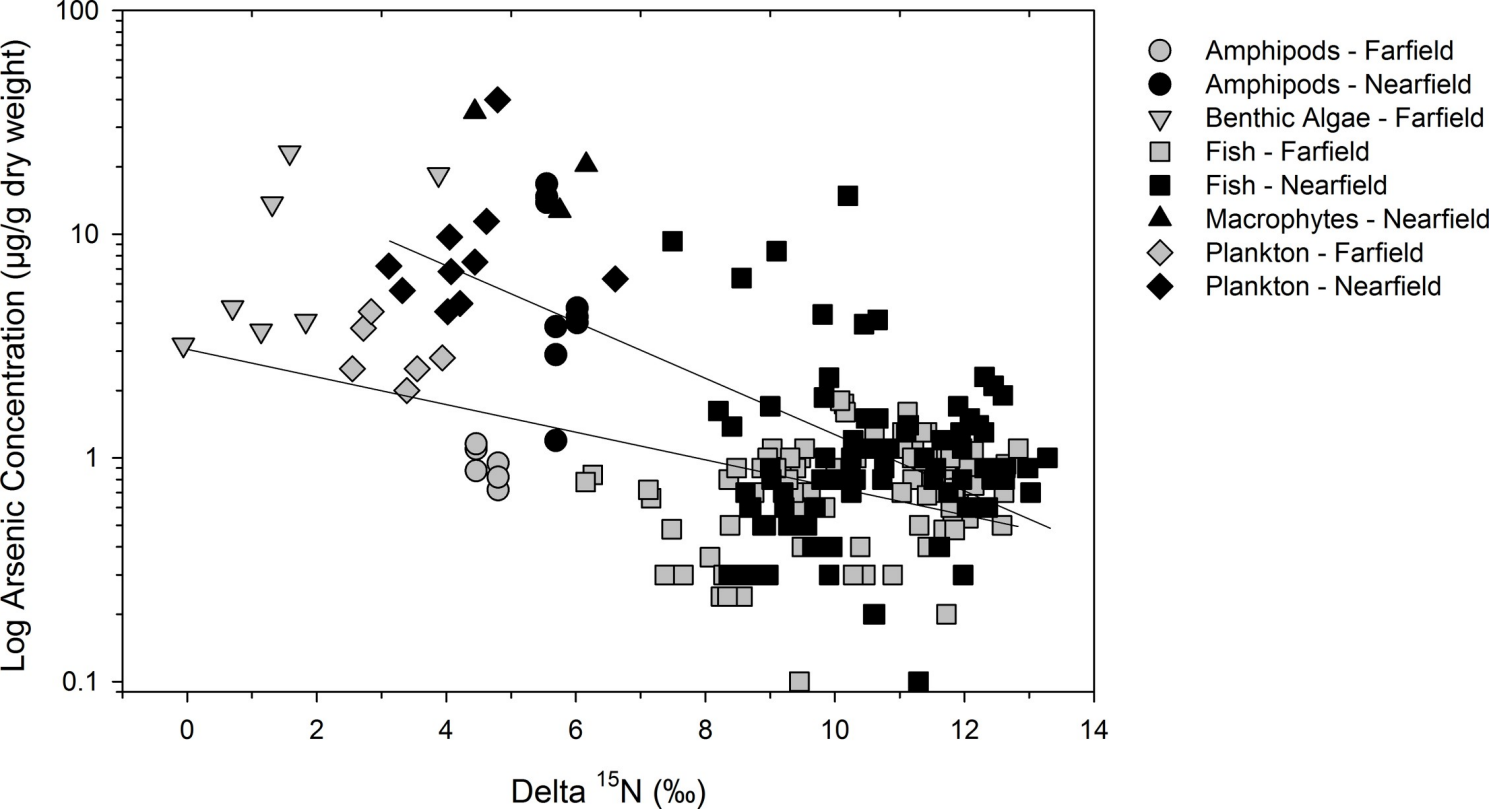
Food web total arsenic concentrations in relation to trophic position (δ^15^N). Lower trophic level biota (benthic primary producers, plankton, and amphipods) and fishes are identified separately, as are data for the nearfield and farfield areas in Great Slave Lake, Northwest Territories, Canada. The GLM was statistically significant (*r*^*2*^ = 0.41, *F* = 51, *p* < 0.001, *n* = 217), and the regression slope for nearfield biota was significantly steeper (*p* < 0.001) than for the farfield area. The y-axis is presented on a logarithmic scale.

Total arsenic concentrations of plankton and amphipods were higher in the nearfield area compared to the farfield (Table 4). Plankton of two mesh sizes (> 64 and > 200 μm) had, on average, three times more total arsenic in the nearfield (two-way ANOVA, *n* = 16, capture area effect: *F* = 14, *p* = 0.003; plankton mesh size effect: *F* = 3.6, *p* = 0.08; interaction term: *F* = 0.2, *p* = 0.669). Similarly, amphipods had eight times higher total arsenic in the nearfield (*t*-test, *p* < 0.001, *n* = 15). Fishes generally had lower total arsenic concentrations than benthic primary producers, amphipods and plankton (Fig 4, Table 4). Biodilution occurred in the food web, as indicated by a decline of log total arsenic concentration with greater δ^15^N values of biota (linear regression: *r*^*2*^ = 0.29, *p* < 0.001, *n* = 217, using arsenic concentrations in fish muscle). A model that accounted for concentration differences between the nearfield and farfield areas indicated that biotic arsenic declined more with trophic position (δ^15^N) in the nearfield area (GLM, *r*^*2*^ = 0.41, *F* = 51, *p* < 0.001, *n* = 217; capture area*δ^15^N interaction: *F* = 14, *p* < 0.001) (Fig 6).

**Table 4:** Total arsenic concentrations in benthic primary producers, profundal amphipods, and plankton in Great Slave Lake, Northwest Territories, Canada.

	Nearfield		Farfield	
Biota	Mean ± SD	*n*	Mean ± SD	*n*
Macrophytes	22.8 ± 11.4	3	n.d.	
Benthic Algae	n.d.		10.2 ± 8.3	7
Plankton (>64 μm)	13.4 ± 13.1	6	3.6 ± 1.0	3
Plankton (>200 μm)	5.8 ± 1.4	4	2.4 ± 0.4	3
Profundal Amphipods	7.4 ± 5.9	9	0.9 ± 0.2	6

Concentrations presented as μg/g dry weight.

n.d. = not determined

The influence of trophic position on biotic total arsenic concentrations was due to differences in bioaccumulation between fish and biota near the base of the food web. When only the fish community was examined, neither liver nor muscle total arsenic concentrations were correlated with fish δ^15^N values (*p* ≥ 0.222). Further, capture area was not a significant influence in the δ^15^N models (GLMs, capture area*δ^15^N interaction, *p* ≥ 0.151, *n* = 176 or 64 for muscle or liver). The analysis of muscle total arsenic concentration in relation to fish δ^15^N included 13 species that ranged in δ^15^N from 6.2–13.2‰.

### Habitat-specific bioaccumulation of arsenic in fishes

**Fig 7:**
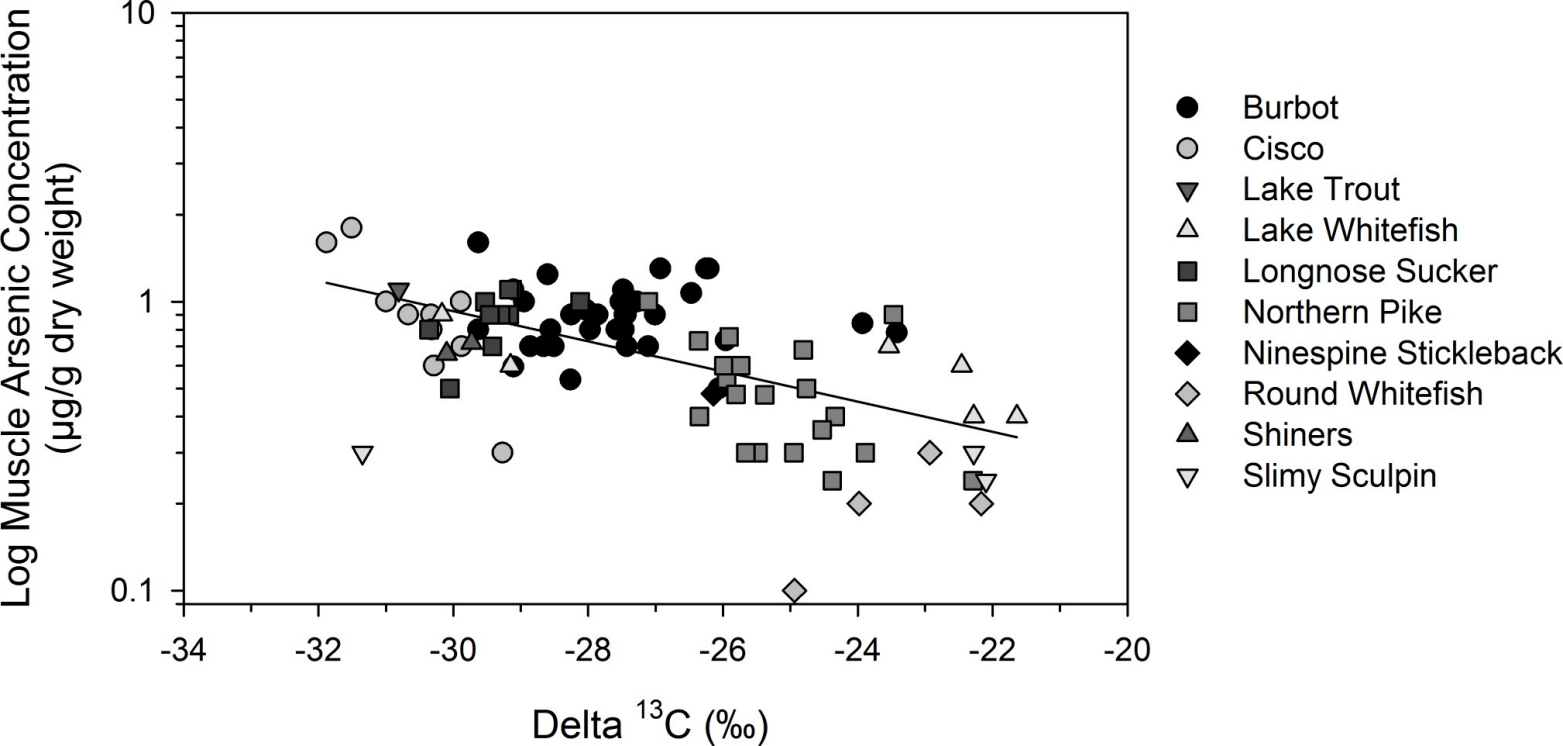
Correlation between muscle total arsenic concentration and carbon source (δ^13^C) in fishes. Data are for the farfield area (*r*^*2*^ = 0.33, *p* < 0.001, *n* = 92) in Great Slave Lake, Northwest Territories, Canada. Total arsenic concentrations are presented on a logarithmic scale, and individual fish species are identified. One muscle sample outlier was not included.

Delta ^13^C values of fishes, which ranged widely from -21.6‰ to -31.9‰, were used to infer dietary carbon sources and habitat-specific feeding. Pelagic-feeding fishes (with more negative ^13^C values) tended to have higher muscle and liver total arsenic concentrations than those that fed in the littoral zone (GLM, *r*^*2*^ = 0.16 or 0.08, *F* = 16 or 7, *p* < 0.01, *n* = 175 or 64 for muscle or liver, 1 muscle sample outlier was not included). For muscle total arsenic concentrations, there was a stronger association with fish δ^13^C in the farfield than in the nearfield (capture area*δ^13^C interaction: *F* = 5, *p* = 0.023) (Fig 7). In the nearfield, small-sized burbot and slimy sculpin with higher total arsenic concentrations were captured nearshore by the effluent outflow at Baker Creek and those fishes had higher δ^13^C values indicative of littoral feeding. The point-source exposure for those littoral fishes may have influenced the δ^13^C correlation in the nearfield. In the farfield area, where there were no point sources of arsenic, species that fed offshore such as cisco, longnose sucker and burbot tended to have higher muscle total arsenic concentrations than littoral feeders (with more positive δ^13^C values) such as lake whitefish, round whitefish and northern pike (Fig 7).

### Comparison of fish arsenic concentrations with other studies

**Fig 8:**
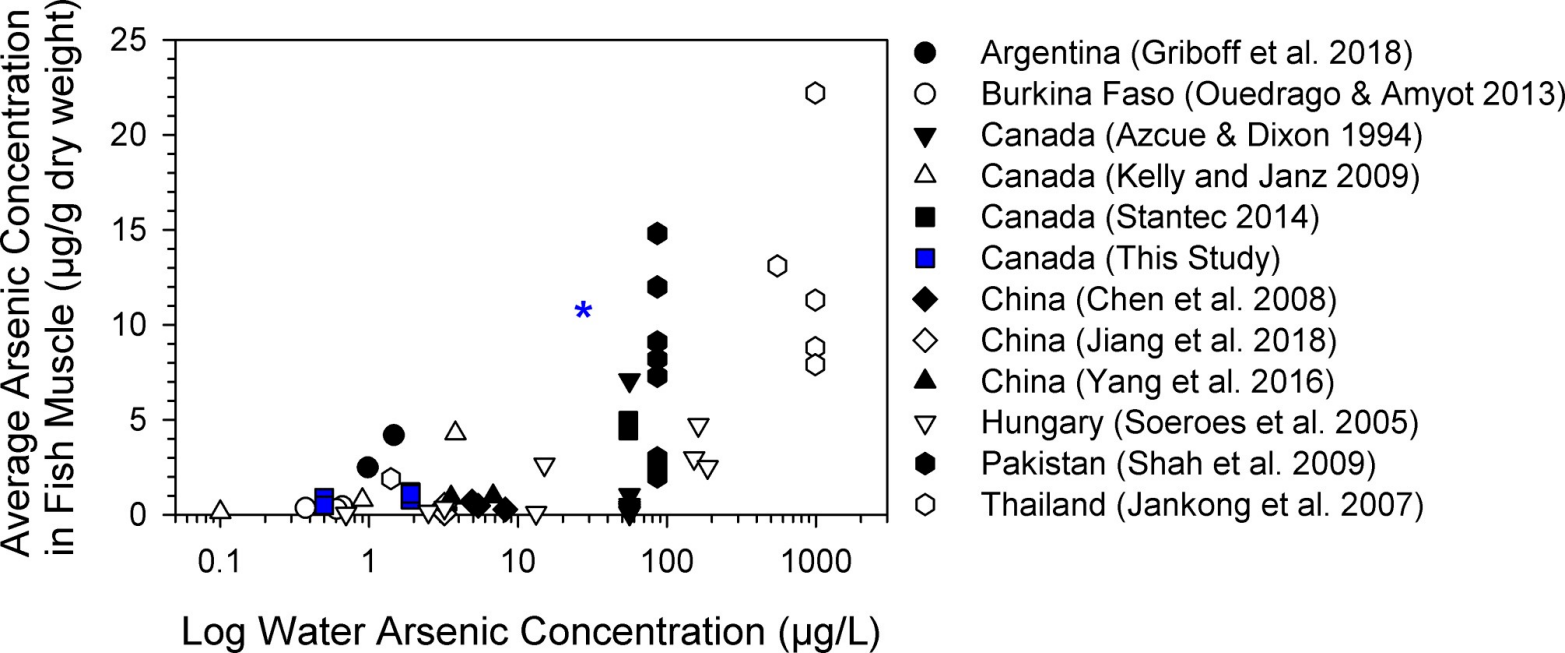
Correlation between total arsenic concentrations in surface water and fish muscle from lakes around the world. Data sources for the 33 lakes and ponds are identified in the figure legend (Spearman *rho* = 0.50, *p* < 0.001, *n* = 67). Mean total arsenic concentrations in burbot, lake whitefish and northern pike from nearfield and farfield areas of Great Slave Lake are identified as blue squares. The mean muscle total arsenic concentration for 13 burbot collected at or near the Baker Creek effluent outflow in the nearfield area is presented separately (blue asterisk).

Water arsenic concentration ranged four orders of magnitude from 0.1–990 μg/L among lakes in the global dataset (Fig 8). Lake-mean concentrations of total arsenic in fish muscle ranged two orders of magnitude from 0.1–22.2 μg/g dry weight and were positively correlated with water arsenic concentration (Spearman *rho* = 0.50, *p* < 0.001, *n* = 67). Considerable inter-species variation in fish total arsenic concentration was observed for some lakes with elevated water arsenic, such as for Manchar Lake in Pakistan [15] and Moira Lake in Canada [13] (Fig 8). Muscle total arsenic concentrations measured in fishes from the nearfield and farfield areas of this study were comparable to other lakes around the world that had similar levels of water arsenic. Burbot collected near the Giant Mine effluent outflow to Yellowknife Bay were presented separately from other burbot in the nearfield area, and their average muscle total arsenic concentration (10.9 μg/g, *n* = 13) was similar to highly contaminated sites with water arsenic > 100 μg/L [15, 53, 54].

## Discussion

### Arsenic accumulation in relation to other study elements

Total concentrations of arsenic in burbot, lake whitefish, and northern pike differed from the accumulation of three metals (copper, manganese, zinc) released from mining, as indicated by the RDA results (Fig 2). Arsenic was more contaminated in the surface water and sediment of Yellowknife Bay compared with copper, manganese and zinc [30, 55], and therefore, higher exposure and greater spatial variability in environmental concentrations of arsenic likely played a role. In addition, copper, manganese and zinc are essential elements, in contrast with arsenic, and those elements may undergo greater homeostatic regulation by fish resulting in lower variability of concentrations among individuals [56]. Antimony, lead, and silver showed little bioaccumulation and were predominately below analytical detection in fish tissues (Table 1). In the case of silver, concentrations were above analytical detection in liver but not muscle. Other research similarly found low concentrations of antimony [22], lead [21], and silver (in muscle) [57] in freshwater fishes from contaminated sites in temperate ecosystems.

### Arsenic bioaccumulation in the nearfield area

Average total arsenic concentrations in muscle of burbot, lake whitefish, and northern pike from the nearfield area were higher than the farfield, consistent with 3 to 5 times higher arsenic in surface waters at the north end of the bay compared to the farfield area [30]. Arsenic enrichment of 3 to 8 fold was observed for plankton and amphipods in the nearfield area (Table 3), while fish muscle total arsenic concentrations were approximately two-fold higher. Lower enrichment in fish tissue compared with other food web components is likely due to inefficient trophic transfer of arsenic (biodilution). Arsenic in nearfield surface waters was relatively low (mean ± SD = 3.1 ± 0.5 μg/L in summer) [30], but still high enough to detect enhanced arsenic bioaccumulation in the food web relative to the farfield (<1 μg/L in summer) [30]. Although an impact from mining contamination was detected, the total arsenic concentrations in the majority of fish were relatively low, and a recent human health risk assessment determined that the consumption of country food (including fish from Yellowknife Bay) posed a negligible to very low risk of health impacts to local residents [58].

Total concentrations of 2–6 μg/g wet weight (10–30 μg/g dry weight) in fish tissue are associated with a chronic response to arsenic exposure [1]. Small-sized burbot collected near the effluent outflow from Baker Creek were the only fish with concentrations that approached or exceeded a threshold of 10 μg/g dry weight. Environmental effects monitoring found that slimy sculpin collected at Baker Creek had higher total arsenic concentrations (mean of 1.3 μg/g wet weight or 6.5 μg/g dry weight), reduced body condition, and larger livers compared to a reference site [35, 59]. Further research is warranted to investigate potential chronic toxicity of arsenic exposure for juvenile burbot with elevated body burdens in the nearfield study area.

### Patterns of arsenic in fish liver and muscle

Arsenic accumulated differently in muscle and liver, with higher total concentrations in liver than muscle for burbot and lake whitefish, but lower arsenic in liver than muscle for northern pike. In a review of arsenic bioaccumulation in fishes, Kumari et al. [60] found considerable variation among studies in the tissue that was reported as having the highest arsenic concentration. Spatial and temporal comparisons in this study also showed different arsenic concentration patterns between tissues, with greater differences in muscle than liver between nearfield and farfield areas, and temporal change in liver but not muscle over a 20-year period in the nearfield.

The different patterns of arsenic accumulation in liver and muscle may be due to several processes. In fish, arsenic from ingested prey is transported from the intestine into the blood stream and then distributed to the liver and remaining body including muscle [8]. The liver is an organ that filters blood and plays a role in the detoxification and elimination of arsenic, while muscle acts as a site of accumulation [8, 61]. In that context, liver arsenic concentrations are representative of arsenic exposure for fish, while muscle concentrations reflect retention in the body [61, 62]. Fish species differ in their arsenic absorption efficiencies in the intestine and elimination rates from the body [12, 61, 63], which may have contributed to the different ratios of liver to muscle arsenic among species. Further, species-specific accumulation of arsenic has been linked to the extent of arsenic biotransformation in fish [20]. Fish metabolize ingested inorganic arsenic from prey into organic arsenic (such as dimethylarsenic acid and arsenobetaine), and this biotransformation results in accumulation in the muscle [12, 64]. Arsenic speciation of fishes and their diet were not determined in this study, and future measurements may help to explain the bioaccumulation patterns. Earlier work on arsenic in fishes of Yellowknife Bay showed arsenic was predominately as organic species such as arsenobetaine and dimethylarsinic acid rather than inorganic arsenic [35–37]. Those organoarsenicals may originate from the transformation of inorganic arsenic to arsenosugars and subsequently to arsenobetaine by freshwater plankton, although less information is available on these processes than in the marine environment [65].

Arsenic exposure varied within and among fish species. Muscle and liver arsenic concentrations were higher in fishes that fed more in the pelagic environment (more negative δ^13^C values) (Fig 7). In the farfield, burbot–which are obligate piscivores as adults [66] and feed primarily on pelagic-based prey–had higher arsenic concentrations than lake whitefish and northern pike, which are littoral feeders [67]. In addition, fish movement may have influenced arsenic exposure (in particular, arsenic in the liver of burbot and lake whitefish) if some individuals moved between the nearfield and farfield areas. Burbot tend to move into Yellowknife Bay around January to spawn, retreating by April [68]. Likewise, lake whitefish move large distances in great lakes [69] and, within the study area, migrate in autumn from the main body of Great Slave Lake into the Yellowknife River at the north end of Yellowknife Bay. Large northern pike are mobile predators and can move several kilometres to feed [70]. Despite the potential confounding factor of fish movement, significant differences in arsenic concentrations were observed for all three species between the nearfield and farfield areas.

Lower total arsenic concentrations were found in liver of the more recently collected fishes compared to those collected by Jackson et al. [34], indicating lower arsenic exposure after closure of the Giant Mine. Surface water arsenic concentrations in the nearfield area of Yellowknife Bay slightly declined from 4.7 ± 0.5 μg/L in 1992–1993 (mean ± SE, *n* = 18) [34] to 3.1 ± 0.5 μg/L in 2014–2015 (*n* = 22) [30]. Muscle total arsenic concentrations remained the same between the two periods for all three species. The small decline in water arsenic may have been insufficient to alter accumulation in fish muscle.

### Influence of fish size and age

Several lines of evidence indicate that bioaccumulation of arsenic in fishes was not affected by size or age. Inconsistent correlations between total arsenic concentrations and size or age were found (positive, negative, no correlation) (Table 3). In some cases, size did not have the same influence for that species in the nearfield and farfield areas (Fig 3). Therefore, the correlation with size was likely due to differences in arsenic exposure among size classes rather than an effect of bioaccumulation over time. In the case of burbot, smaller-sized juveniles with higher arsenic in the nearfield area (top panel, Fig 3) were captured in the vicinity of the effluent outflow for Giant Mine and were likely exposed to higher arsenic levels compared with larger individuals that resided in less impacted areas of the nearfield. Although size and age effects on arsenic concentrations in fishes have been reported in the literature [19, 71], the results from this study are consistent with findings that arsenic is eliminated relatively quickly from fish [8, 61, 72]. Arsenic bioaccumulated more in the higher exposure nearfield area, but no evidence was found for long-term increases with age for long-lived, slow growing subarctic fishes in this study.

### Food web bioaccumulation of arsenic

Arsenic biodilution occurred with increasing trophic position in the food web (Fig 6), although the differences in arsenic accumulation were between fish and aquatic biota at lower trophic levels. Total arsenic concentrations in fish species that spanned two trophic levels were not influenced by trophic position, similar to findings of Ward et al. [73] in lakes of New England, USA. Those authors suggested there are fundamental differences in arsenic bioaccumulation between invertebrates and fish that may result in higher concentrations in invertebrates, for example due to differences in exposure routes or arsenic binding to the chitin exoskeleton of invertebrates [73]. Prey type (i.e. invertebrate or fish) did not appear to affect arsenic concentrations in fishes of this study because lake whitefish (a benthivore) and northern pike (a piscivore) had similar concentrations of arsenic despite holding markedly different food web positions [67].

Pelagic-feeding fishes tended to have higher total arsenic concentrations than littoral feeders, particularly in the farfield area (Fig 7). Note that several fish species (including cisco, round whitefish, and longnose sucker) were only captured in the farfield area, which may explain the stronger correlation between tissue total arsenic concentrations and carbon stable isotope values. The influence of habitat-specific feeding has been observed for fish species of temperate lakes in the USA and China [18, 21]. It remains unclear what environmental process was driving this ecological variation. Habitat differences in water arsenic exposure may have played a role if concentrations were higher in pelagic relative to littoral waters. Another possible explanation is that arsenic speciation varied between plankton, littoral algae and macroinvertebrates, and those differences in speciation affected arsenic retention in fishes. Organic arsenic (particularly arsenobetaine) in the diet of fish is more efficiently retained than inorganic arsenic [20], and the composition of organic arsenic compounds and the proportion of inorganic arsenic in biota near the base of the food web can vary considerably [74, 75]. In Yellowknife, zooplankton from mine-impacted lakes had variable proportions of inorganic and organic arsenic, and the highest proportion of organic arsenic (including arsenobetaine) was found in zooplankton from the least contaminated lake [65].

### Comparison of subarctic fishes with other lakes worldwide

Subarctic fishes from this study accumulated arsenic to similar levels as fishes from lakes around the world including temperate and tropical regions (Fig 8). This preliminary meta-analysis suggests that empirical models relating water arsenic exposure to fish tissue burdens may be broadly applicable across different types of lentic ecosystems. Arsenic has no biomagnification potential and there were no long-term increases in bioaccumulation with fish age. Thus, the concentrations in fishes from Great Slave Lake (including older, large-bodied fish) were consistent with productive waterbodies that had similar water arsenic concentrations. This pattern contrasts with other contaminants that have high potential for bioaccumulation and biomagnification such as mercury and organochlorines, to which Arctic freshwater fishes can be sensitive because of slow growth rates, older ages of populations, and (sometimes) longer food chains [76, 77]. Total arsenic concentrations in muscle of freshwater fishes in the Canadian Arctic are generally < 0.5 μg/g dry weight [78, 79], although those studies were not included in this meta-analysis due to a lack of water arsenic data. Further work is warranted to develop a comprehensive global dataset that includes more sites and additional explanatory factors such as arsenic speciation and concentrations in sediment and prey.

## Conclusions

This first study of arsenic accumulation in a subarctic freshwater food web found increased arsenic exposure to fishes from gold mining contamination on the shores of Yellowknife Bay, Great Slave Lake. Differences in fish total arsenic concentrations were observed between nearfield and farfield areas, and over a 20-year period, despite a relatively small gradient in surface water arsenic. Total arsenic concentrations in fishes were tissue and species-specific. Biodilution of arsenic occurred in the food web, and habitat-specific feeding partly explained the arsenic accumulation in different fish species. Overall, larger and older fishes commonly harvested in Great Slave Lake for human consumption did not have higher arsenic than smaller and younger fishes. Water arsenic concentrations broadly explained levels of arsenic accumulation in fishes from published studies of lakes and ponds around the world. These findings on the importance of spatial variation in water exposure, biodilution, and habitat-specific trophic transfer for determining arsenic concentrations in fishes improve our understanding of bioaccumulation responses to mining contamination of arsenic in freshwater food webs.

## Supporting information

S1 Text Supplemental information on the data analysis. (PDF)
