# Supplementary material for: Arsenic bioaccumulation in subarctic fishes of a mine-impacted bay on Great Slave Lake, Northwest Territories, Canada
Source: PLoS One. 2019 Aug 23;14(8):e0221361. doi: 10.1371/journal.pone.0221361 (PMC6707560; doi:10.1371/journal.pone.0221361)
Supplement: S1 Text — (PDF) [file pone.0221361.s001.pdf]

Supplemental information for:

Arsenic bioaccumulation in Subarctic fishes of a mine-impacted bay on Great Slave Lake, Northwest Territories, Canada

John Chételat*[1], Peter A. Cott[2,#a], Maikel Rosabal[3], Adam Houben[4,#b] Christine McClelland[1], Elise Belle Rose[5], and Marc Amyot[6]

[1] Environment and Climate Change Canada, National Wildlife Research Centre, Ottawa, ON, Canada

[2] Environment and Natural Resources — Cumulative Impact Monitoring Program, Government of the Northwest Territories, Yellowknife, NT X1A 2L9, Canada

[3] Département des sciences biologiques, Université de Quebec à Montréal, Montreal, QC H2X 1Y4, Canada

[4] Consultant, 1-46 Rue Laurel, Ottawa, ON K1Y 3C9, Canada

[5] Geography and Environmental Studies, Carleton University, Ottawa, ON K1S 5B6, Canada

[6] Centre d’études nordiques (CEN), Département de sciences biologiques, Université de Montréal, Montréal, QC H2V 2S9, Canada

[#a] Current address: Department of National Defence, Joint Task Force North, 4816 49 St., Yellowknife, NT X1A 2R3, Canada

[#b] Current address: Science and Technology Division, Polar Knowledge Canada, 1 Uvajuq Road, Cambridge Bay, NU X0B 0C0, Canada

*Corresponding author

Email: john.chetelat@canada.ca

Data analysis

Conversion of whole-body to muscle concentrations:

Whole-body element concentrations (arsenic, copper, manganese, zinc) of small-bodied fish (n = 24) were converted to muscle concentrations using the following measured factors for the ratio of muscle:whole-body concentration (mean ± 95% confidence interval): arsenic = 0.60 ± 0.09, copper = 0.58 ± 0.12, manganese = 0.15 ± 0.06, zinc = 0.29 ± 0.06). Whole-body to muscle conversions were not made for antimony, lead and silver because concentrations were below analytical detection.

Compilation of fish datasets for Yellowknife Bay:

Fish data generated in this study were combined with element concentrations in fish of the study area reported in the grey literature and a published paper to conduct a more comprehensive analysis of metal(loid) accumulation in fish. Fish size, age, and element concentrations in muscle and liver were included from Stantec (2014a) for 7 burbot, 84 lake whitefish, and 104 northern pike that were captured in 2012–2013. Data were included for 7 burbot and 17 lake whitefish captured by Cott et al. (2016) in 2011. Age estimates were not available for fish from Cott et al. (2016) and for burbot from Stantec (2014a). Data from an older report (Jackson et al. 1996) on fish collections in Yellowknife Bay in 1992–1993 were included to examine temporal variation in arsenic concentrations. Fish length, age and arsenic concentrations of liver and muscle in 11 burbot, 128 lake whitefish, and 21 northern pike were available from Jackson et al. (1996). The general locations of capture sites for fish from the literature datasets are presented in Figure 1. Details of the capture and analytical methods can be found in the respective reports. Fish element

concentrations are reported on a dry weight basis in this paper. For data from Stantec (2014a), element concentrations were converted to dry weight values using the percent moisture content measured on individual samples. For Jackson et al. (1996), element concentrations were converted to dry weight values using the average percent moisture of muscle (80%) and liver (78%) reported in Stantec (2014a). Half the detection limit was used for non-detect values.

Comparison of fish arsenic bioaccumulation in Yellowknife Bay with other studies

A literature search was performed using the abstract and citation database Scopus (Elsevier B.V.) to compile and compare published field studies around the world where arsenic concentrations were measured in both water and fishes of lakes or ponds. Water arsenic concentration was used as an indicator of ecosystem exposure to the food chain. The dataset consisted of 67 records of average arsenic concentrations in fish muscle (μg/g dry weight) from 33 waterbodies, which were obtained from ten published papers (Azcue and Dixon 1994; Chen et al. 2008; Griboff et al. 2018; Jankong et al. 2007; Jiang et al. 2018; Kelly and Janz 2009; Ouédraogo and Amyot 2013; Shah et al. 2009; Soeroes et al. 2005; Yang et al. 2017), a consultant report for an adjacent lake impacted by the Giant Mine (Lower Martin Lake, Stantec 2014a), and this study. Where possible, mean concentrations of individual fish species from the same waterbody were included as separate data entries. Water arsenic concentrations were obtained from Chételat et al. (2018) for the nearfield and farfield areas of Yellowknife Bay, from INAC (2018) for the effluent outflow site in Yellowknife Bay, from Stantec (2014b) for Lower Martin Lake, and from Kelly and Janz (2008) for the fish arsenic data from Kelly and Janz (2009). Muscle arsenic concentrations were converted from a wet to dry weight basis for three

studies (assuming 80% moisture content) and from whole body to muscle for one study (assuming a factor of 0.6).

References

Azcue, J.M., and Dixon, D.G., 1994. Effects of past mining activities on the arsenic concentration in fish from Moira Lake, Ontario; *Journal of Great Lakes Research*,Volume 20, p. 717-724.

Chen, C.Y., Pickhardt, P.C., Xu, M.Q., and Folt, C.L., 2008. Mercury and arsenic bioaccumulation and eutrophication in Baiyangdian Lake, China; *Water, Air, and Soil Pollution*,Volume 190, p. 115-127.

Cott, P.A., Zajdlik, B.A., Palmer, M.J., and McPherson, M.D., 2016. Arsenic and mercury in lake whitefish and burbot near the abandoned Giant Mine on Great Slave Lake; *Journal of Great Lakes Research*,Volume 42, p. 223-232.

Griboff, J., Horacek, M., Wunderlin, D.A., and Monferran, M.V., 2018. Bioaccumulation and trophic transfer of metals, As and Se through a freshwater food web affected by antrophic pollution in Córdoba, Argentina; *Ecotoxicology and Environmental Safety*,Volume 148, p. 275-284.

Jackson, F.J., Lafontaine, C.N., and Klaverkamp, J., 1996. *Yellowknife - Back Bay Study on Metal and Trace Element Contamination of Water, Sediment and Fish,* Joint Report of Indian and Northern Affairs Canada with Fisheries and Oceans Canada.

Jankong, P., Chalhoub, C., Kienzl, N., Goessler, W., Francesconi, K.A., and Visoottiviseth, P., 2007. Arsenic accumulation and speciation in freshwater fish living in arsenic-contaminated waters; *Environmental Chemistry*,Volume 4, p. 11-17.

Jiang, Z., Xu, N., Liu, B., Zhou, L., Wang, J., Wang, C., Dai, B., and Xiong, W., 2018. Metal concentrations and risk assessment in water, sediment and economic fish species with various habitat preferences and trophic guilds from Lake Caizi, Southeast China; *Ecotoxicology and Environmental Safety*,Volume 157, p. 1-8.

Kelly, J.M., and Janz, D.M., 2008. Altered energetics and parasitism in juvenile northern pike (*Esox lucius*) inhabiting metal-mining contaminated lakes; *Ecotoxicology and Environmental Safety*,Volume 70, p. 357-369.

Kelly, J.M., and Janz, D.M., 2009. Assessment of oxidative stress and histopathology in juvenile northern pike (*Esox lucius*) inhabiting lakes downstream of a uranium mill; *Aquatic Toxicology*,Volume 92, p. 240-249.

Ouédraogo, O., and Amyot, M., 2013. Mercury, arsenic and selenium concentrations in water and fish from sub-Saharan semi-arid freshwater reservoirs (Burkina Faso); *Science of the Total Environment*,Volume 444, p. 243-254.

Shah, A.Q., Kazi, T.G., Arain, M.B., Jamali, M.K., Afridi, H.I., Jalbani, N., Baig, J.A., and Kandhro, G.A., 2009. Accumulation of arsenic in different fresh water fish species - potential contribution to high arsenic intakes; *Food Chemistry*,Volume 112, p. 520-524.

Soeroes, C., Goessler, W., Francesconi, K.A., Kienzl, N., Schaeffer, R., Fodor, P., and Kuehnelt, D., 2005. Arsenic speciation in farmed Hungarian freshwater fish; *Journal of Agricultural and Food Chemistry*,Volume 53, p. 9238-9243.

Stantec, 2014a. *Analysis of Contaminants in Tissues of Fish Captured in the Yellowknife Bay Area, NT*, Yellowknife, NT, Prepared by Stantec Consulting Ltd. for Public Works and Government Services Canada.

Stantec, 2014b. *Aquatic Data Collection in Lower Martin Lake, Upper Baker Creek and Trapper Creek. Prepared for: Public Works and Government Services Canada*, Yellowknife, NT.

Yang, F., Zhang, N., Wei, C., Liu, J., and Xie, S., 2017. Arsenic speciation in organisms from two large shallow freshwater lakes in China; *Bulletin of Environmental Contamination and Toxicology*,Volume 98, p. 226-233.
